# Recommended Mass Spectrometry-Based Strategies to Identify Ricin-Containing Samples

**DOI:** 10.3390/toxins7124854

**Published:** 2015-11-25

**Authors:** Suzanne R. Kalb, David M. Schieltz, François Becher, Crister Astot, Sten-Åke Fredriksson, John R. Barr

**Affiliations:** 1 Centers for Disease Control and Prevention, 4770 Buford Hwy NE, Atlanta, GA 30341, USA; skalb@cdc.gov (S.R.K.); dschieltz@cdc.gov (D.M.S.); 2 Service de Pharmacologie et d’Immunoanalyse, Institut de Biologie et de Technologies de Saclay (iBiTec-S), Commissariat à l’Énergie Atomique et aux Énergies Alternatives (CEA), 91191 Gif-sur-Yvette, France; francois.becher@cea.fr; 3 The Swedish Defence Research Agency (FOI), SE-901 82 Umeå, Sweden; crister.astot@foi.se (C.A.); sten-ake.fredriksson@foi.se (S.-A.F.)

**Keywords:** ricin, RCA120, *Ricinus communis*, mass spectrometry

## Abstract

Ricin is a protein toxin produced by the castor bean plant (*Ricinus communis*) together with a related protein known as *R. communis* agglutinin (RCA120). Mass spectrometric (MS) assays have the capacity to unambiguously identify ricin and to detect ricin’s activity in samples with complex matrices. These qualitative and quantitative assays enable detection and differentiation of ricin from the less toxic RCA120 through determination of the amino acid sequence of the protein in question, and active ricin can be monitored by MS as the release of adenine from the depurination of a nucleic acid substrate. In this work, we describe the application of MS-based methods to detect, differentiate and quantify ricin and RCA120 in nine blinded samples supplied as part of the EQuATox proficiency test. Overall, MS-based assays successfully identified all samples containing ricin or RCA120 with the exception of the sample spiked with the lowest concentration (0.414 ng/mL). In fact, mass spectrometry was the most successful method for differentiation of ricin and RCA120 based on amino acid determination. Mass spectrometric methods were also successful at ranking the functional activities of the samples, successfully yielding semi-quantitative results. These results indicate that MS-based assays are excellent techniques to detect, differentiate, and quantify ricin and RCA120 in complex matrices.

## 1. Introduction

Ricin is a protein toxin produced by the castor bean plant, *Ricinus communis*. It is composed of two polypeptide chains, known as the A- and B-chains, which are each approximately 32 kDa, making intact ricin approximately 64 kDa with glycosylation [1]. Ricin belongs to the type 2 ribosome-inactivating protein toxins (RIP-II toxins). The A-chain of ricin has a specific enzymatic activity, as it depurinates a single adenosine that is part of a GAGA tetraloop of 28S ribosomal RNA [2,3]. This prevents the binding of elongation factor 2 (EF-2) to the ribosome, leading to the cessation of protein synthesis [4]. The termination of protein synthesis causes the severe physical effects of the ricin toxin and can lead to death. The B-chain has lectin activity and brings the enzymatically active A-chain to its target through cell receptor binding and endocytosis [5]. Both the A and B-chains contain *N*-linked glycosylation [6,7], and although both chains are needed for *in vivo* toxicity, glycosylation of only the B-chain is needed for maximum toxicity [8].

**Figure 1:**
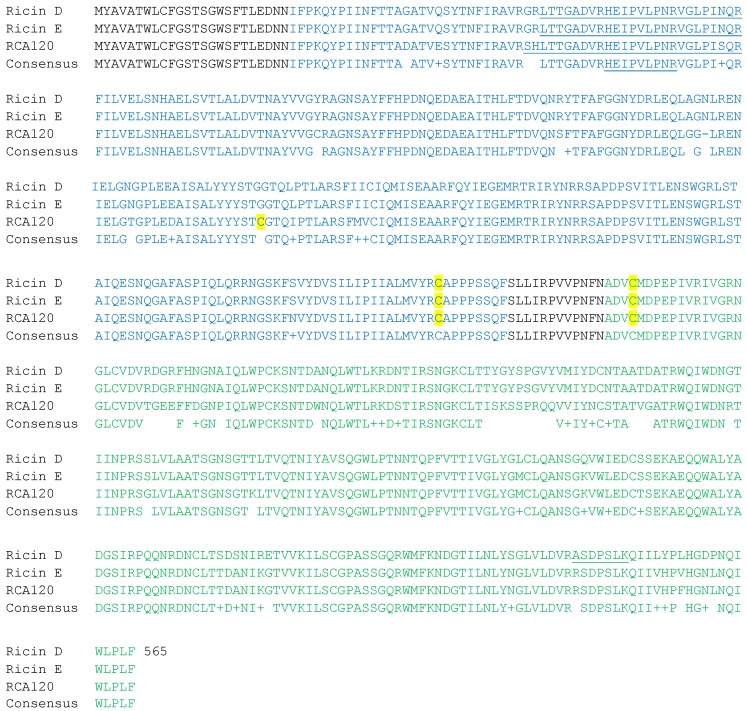
Amino acid sequence alignment of ricin D, ricin E and RCA120. Amino acids which differ are noted in the consensus line as an empty space or as “+” if the changes are conserved. The A-chain is depicted in blue and the B-chain in green. Peptides used for targeted mass spectrometric identification/differentiation are underlined. Highlighted cysteines indicate disulfide bond formation.

*R. communis* also produces a closely related protein known as RCA120, or agglutinin. RCA120 is a heterodimer with two A-chains and two B-chains linked by one disulfide bond between Cys156 of each A-chain and has an intact mass of approximately 120 kDa. It has the same enzymatic activity as ricin, but is orders of magnitude less active than ricin [9]. These two proteins are approximately 89% identical in amino acid sequence, as seen in Figure 1, which makes the two proteins quite similar; in fact, many antibodies have cross-reactivity between ricin and RCA120 [10]. Nonetheless, the 11% difference can be used for differentiation between the two proteins, provided that an appropriate analytical technique is utilized.

**Table 1:** Identity of EQuATox samples with qualitative results using mass spectrometric (MS/MS) analysis. Signature trypsin digest peptides of ricin D (Uniprot P02879).

Sample #		S1	S2	S3	S4	S5	S6	S7	S8	S9
Matrix		0.1% BSA in PBS	0.1% BSA in PBS	0.1% BSA in PBS	Skimmed milk	0.1% BSA in PBS	0.1% BSA in PBS	0.1% BSA in PBS	Meat extract	Organic fertilizer
Nominal toxin		N/A	RCA120	Ricin	Ricin	RCA120	Ricin	Ricin	Ricin	Ricin, RCA120
Conc.		N/A	572851	504	473	445	589508	0.414	484	306, 42
Unit			μg/L	μg/L	μg/L	μg/L	μg/L	μg/L	μg/L	mg/kg
Observed toxin			RCA120	Ricin	Ricin, RCA120	RCA120	Ricin	Ricin	Ricin	Ricin, RCA120
Observed toxin activity		−	+	+	+	+	+	−	+	+
Ricin D (Uniprot P02879) signature trypsin digest peptides										
**T#**	**Amino Acid Sequence**	**S1**	**S2**	**S3**	**S4**	**S5**	**S6**	**S7**	**S8**	**S9**
T2a	QYPIINFTTAGATVQSYTNFR			●	●		●		○	●
T5a	LTTGADVR			■ ●	■ ●	■	■ ●		■ ●	■ ●
T7a	VGLPINQR	■	■	■ ●	■ ●	■	■ ●	■	■ ○	■ ●
T9a	AGNSAYFFHPDNQEDAEAITHLFTDVQNR			●	●	●	●		●	●
T10a	YTFAFGGNYDR			●	●	●	●		○	●
T11a	LEQLAGNLR			●	●	●	●		○	●
T23a	FSVYDVSILIPIIALMVYR						●			●
T3b-T5b	NGLCVDVR (-ss-)FHNGNAIQLWPCK			○			○			○
T6b	SNTDANQLWTLK			●	●	●	●		○	●
T11b	WQIWDNGTIINPR			●	●		●		○	●
T14b-T16b	DNCLTSDSNIR (-ss-) ILSCGPASSGQR			○	○		○			○
T18b	NDGTILNLYSGLVLDVR			○	○		○		○	○
T19b	ASDPSLK		■	■	■				■	■
T20b	QIILYPLHGDPNQIWLPLF			●	●	●	●		●	●

■: Identified by targeted liquid chromatography LC-MS/MS; ○: Identified by non-targeted LC-MS/MS and ●: Confirmed by LC-MS/MS.

In addition, there are two ricin isoforms, D and E, which differ in their B-chain sequence. Ricin E is thought to have evolved through a gene recombination between the ricin D and the RCA120 genes [11]. Thus, the B-chain of ricin E shares its *N*-terminus with ricin D and the *C*-terminus with RCA120 as seen in Figure 1. Ricin E is produced in some of the *R. communis* ecotypes studied and has only been detected together with ricin D. Specific differentiation of the ricin isoforms from RCA120 is critical for forensic or epidemiological purposes, and the close similarity between the proteins has made it hard to produce specific antibodies (Ab) without a high cross-reactivity between the proteins [10]. At least one specific immunoassay based on monoclonal Ab has been reported [12] but the availability has generally been a limiting factor for the use of immunological methods for ricin specific detection. However, the sequence differences in ricin and RCA120 can be monitored by mass spectrometry to allow their identification.

Mass spectrometry (MS) is an instrumental measurement technique with widespread use in life sciences. The MS technique has been described in detail elsewhere [13]. A wide variety of instrumental methods involving MS and MS/MS measurements have been successful in detecting, differentiating and quantifying ricin and RCA120 in complex matrices. The different possible analytical strategies will be described/compiled in the following sections—some of these methods have been applied to the analysis of nine blinded samples supplied as part of the EQuATox international ricin proficiency test [14]. The aim was to test the participating laboratories’ capacity to identify the presence or absence of ricin or RCA120 in the blinded samples, consisting of stabilizing buffer, milk, meat extract and fertilizer as seen in Table 1. Furthermore, for the samples, which were found to contain a toxin, differentiation between ricin and RCA120 was desired. The final goals were to quantify the amount of toxin in the samples and to provide a ranking of functional activity of three specified samples.

## 2. Mass Spectrometric (MS) Techniques for Analysis of Ricin

### 2.1. Determination of Molecular Weight of the Intact Ricin Protein by Mass Spectrometric Analysis

The simplest technique for analysis of ricin by mass spectrometry is to detect the intact ricin protein with an approximate molecular weight of 64 kDa. Thus, the detection of a signal with a mass close to 64 kDa could indicate the presence of ricin. This technique was first reported in 2000 using electrospray mass spectrometry for ricin characterization [15]. Electrospray ionization (ESI) of intact ricin produced multiply charged ions, and when the data were de-convoluted, a pattern of peaks corresponding to several glycoforms with a median molecular weight of approximately 63 kDa was generated. MALDI-TOF mass spectrometry has also been reported for the analysis of intact ricin and this work reported a molecular weight of approximately 62,766 Da [16]. In addition to analysis on purified ricin, matrix assisted laser desorption ionization time-of-flight mass spectrometry (MALDI-TOF/MS) of intact ricin was also reported using crude extracts from castor beans [17].

Although analysis of intact ricin can provide useful information including differentiation between ricin and RCA120, it is not a definitive analysis of ricin, as there are many proteins, which have the same approximate molecular weight as ricin. Additionally, this method suffers from the high limit of detection of concentrations greater than 1 μg/mL [17]. Nonetheless, in combination with other detection methods with higher specificity, this analysis can provide useful information.

### 2.2. MS Analysis of the Digested Ricin Protein

Another mass spectrometric technique for ricin identification is peptide mass fingerprinting (PMF) in which the ricin is digested into peptides before analysis by high resolution MS, yielding accurate mass data of the peptides. This allows for protein identification based on the match of acquired peptide masses with the theoretical peptide masses derived *in-silico*. The first application of peptide mass fingerprinting to identify ricin was reported in 2001 [16] where ricin digested with trypsin was analyzed with both MALDI-TOF MS and ESI. Eleven peaks corresponding to the A- and B-chains or ricin were detected by ESI mass spectrometry, and fourteen were visible by MALDI-TOF MS. A similar study in 2006 generated comparable results, with 17 peptides originating from ricin [18], and another study in 2008 reported observation of 20 peptides with a limit of detection of 50 ng/mL [19]. The digestion process was significantly shortened first in 2008 [19] and then in 2010 using on-target tryptic digestion of 30 min to generate 16 peptides from ricin, and some of the peptides could be used to differentiate ricin from RCA120 [20].

Although PMF analyses produce useful information with the potential to differentiate between ricin and RCA120, it is not a conclusive analysis of ricin, as the molecular weight of the peptides carries no information as to their amino acid sequences. In conjunction with other detection methods, PMF can yield useful information in a short timeframe, and the information obtained from this type of analysis can be more informative and is certainly more sensitive than measurements on the intact protein.

## 3. MS/MS Techniques for Analysis of Ricin

### 3.1. Targeted MS/MS Approach

In order to increase the selectivity and sensitivity of the peptide analysis described above, tandem mass spectrometry has been used in targeted approaches for the detection of specific peptides. The operation of triple quadrupole or ion trap mass spectrometers to monitor specific transitions after collision induced dissociation (CID) from parent peptide ions selected in the first MS stage, to product (fragment) ions selected in the second MS stage, allows an analysis of ricin peptides with much higher selectivity than the MS-methods described above. This type of approach is highly targeted and generally achieves detection of a peptide at a lower limit of detection than other types of mass spectrometers, as seen in a 2011 report in which this approach was used to analyze ricin spiked into complex matrices [21]. By focusing on both the A-chain and the B-chain peptides with amino acid sequences unique for ricin, a selective method with an extraordinarily low limit of detection of 0.64 ng/mL was achieved. This approach was later reported for the study of ricin in 18 different *R. communis* cultivars [22].

Targeted MS/MS of ricin was used by at least one laboratory as part of the EQuATox study, and the ricin protein was found in samples S3, S4, S6, S8 and S9 as seen in Table 1. These data show that the targeted MS/MS analysis of ricin was correct, with the exception of the lowest level sample (S7) which was below the detection limit of the method, and that this analytical approach yielded excellent qualitative results for ricin. This process was also used to determine the presence of RCA120 in the nine blinded samples. From these analyses, it was determined that RCA120 was present in samples S2, S4, S5 and S9, as seen in Table 2. It was later revealed that sample S4 contained only ricin and did not contain RCA120. Because RCA120 is several orders of magnitude less toxic than ricin, there has been more of an emphasis on detection of ricin rather than RCA120. Entities have chosen to develop and validate assays for ricin detection, often including RCA120 detection as an afterthought.

Nonetheless, a targeted MS/MS approach remains one of the best methods to detect and differentiate ricin and RCA120 with the ability to distinguish even between ricin D and ricin E, provided that the correct peptide ions are selected for monitoring.

### 3.2. Proteomic MS/MS Approach

The use of scanning tandem mass spectrometers (e.g., quadrupole-time-of-flight (QTOF) MS) allows for the detection of a full spectrum of product ions from a peptide parent ion selected for MS/MS analysis, as described above. As peptide ions can be induced to systematically fragment along the peptide backbone inside the tandem mass spectrometer, the formation of the fragment ions is a controlled process, allowing for determination of amino acid sequences by examining the mass differences of the fragment ions [23]. The use of liquid chromatography (LC) retention time, mass of intact peptide and masses of fragment ions can be used in concert to verify the presence of a particular peptide, which can then be used to verify the existence of a particular protein, provided that the amino acid sequence of that peptide is unique.

MS/MS amino acid sequencing of ricin was first reported in 2005 [24]. In this work, ricin was extracted from castor beans and digested with trypsin with the resultant tryptic peptides analyzed with a QTOF mass spectrometer. This yielded fourteen peptides from the A-chain of ricin and sixteen peptides from the B-chain, in addition to peptides specific for RCA120 [22]. Another similar method includes a shortened digestion time without prior reduction of disulfide bonds, in which the peptides from the A-chain, peptides from the B-chain and the peptide containing the intact interchain disulfide bond verifying the presence of the link between the A- and B-chains were identified and differentiated from RCA120 [25]. This approach was reported for the identification of ricin spiked into complex matrices such as milk, apple juice, serum and saliva [26], in surface swabs from a public health investigation [27], as well as identification of ricin and abrin in a forensic investigation [28].

**Table 2:** RCA120 trypsin digest peptides (chain A: Uniprot P06750, chain B: GI: 225114). Peptides distinguishing RCA120 from ricin are shown in bold type face. Peptides in italics are also found in ricin E (GI: 225419).

Sample #		S1	S2	S3	S4	S5	S6	S7	S8	S9
T2a	**QYPIINFTTADATVESYTNFIR**		●			●				●
T4a	**SHLTTGADVR**		■	■	■	■	■		■	■
T5a	HEIPVLPNR		■ ●	■ ●	■ ●	■ ●	■ ●	■	■ ○	■ ●
T6a	**VGLPISQR**		■ ●	■	■	■ ●	■	■	■	■ ●
T9a	**LEQLGGLR**		●			●				○
T12a	FQYIEGEMR		●	●	●	●	●		○	●
T17a	SAPDPSVITLENSWGR		●	●	●	●	●		○	●
T18a	LSTAIQESNQGAFASPIQLQR		●	●	●	●	●		○	●
T22a-T1b	CAPPPSSQF(ss-) ADVCMDPEPIVR		●		○	●	●			●
T3b	**NGLCVDVFGEEFTDGNPIQLWPCK**		○			○				○
T4b	**SNTDWNQLWTLR**		●			●				●
T8b-T9b	**CLTISK(-ss-) SSPGQQVVIYNCSTATVGATR**		○			○				
T10b	**WQIWDNR**		○			○				○
T11b	**TIINPTSGLVLAATSGNSGTK**		●			●				●
T14b	AEQQWALYADGSIRPQQNR		○				○			○
*T15b-T17b*	*DNCLTTDANIK(-ss-) ILSCGPASSGQR*		○	○	○	○	○			○
*T19b*	*NDGTILNLYNGLVLDVR*		○	○	○	○	○			○
*T22b*	*QIIVHPVHGNLNQIWLPLF*		●	●		●	○		●	○

■: Identified by targeted LC-MS/MS; ○: identified by non-targeted LC-MS and ●: confirmed by LC-MS/MS.

**Figure 2:**
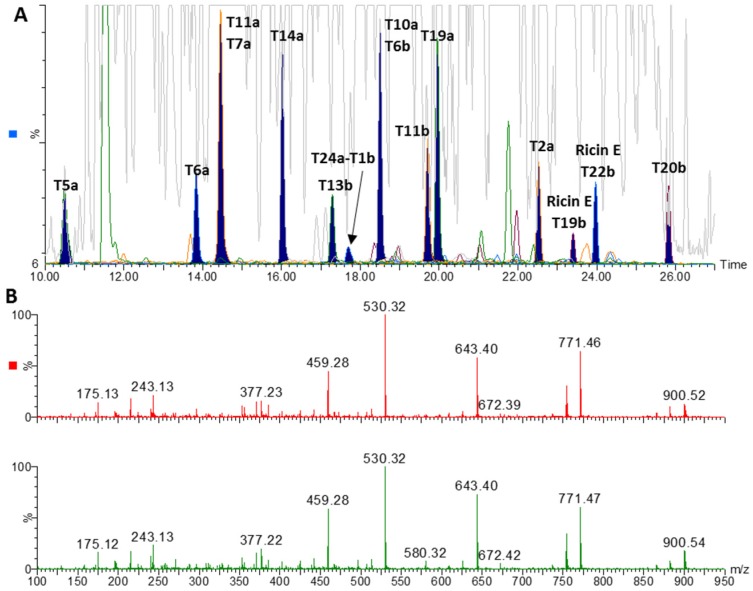
LC-high resolution accurate mass MS analysis of the trypsin digest of a 200 μL aliquot of immunopurified sample S4. (**A**) Base peak chromatogram (grey) overlaid on extracted ion chromatograms of diagnostic ricin D and E peptides (20 ppm mass window). (**B**) Product ion spectrum of *m*/*z* 507.29 at retention time 14.7 min in the S4 digest (upper) compared to the product ion spectrum of T11 of chain A in the ricin reference digest (lower).

Table 1 and Table 2 indicate the peptides detected by targeted LC-MS/MS on a triple quadrupole instrument and by non-targeted LC-high resolution accurate mass MS on a QTOF instrument. The sequence coverage obtained by the non-targeted approach was approximately 70% in the high concentration samples S2 and S6, while in the complex and lower concentration samples S4 and S8 it was 30%–50%. The toxins were unambiguously identified by subsequent analysis by time-scheduled LC-MS/MS of selected peptides indicated in Table 1 and Table 2. Figure 2A shows chromatograms of the LC-MS analysis of the digest of sample S4. Diagnostic ricin peptides are indicated by filled peaks. The offscale peaks in the base peak chromatogram (grey trace) are from trypsin digest peptides of milk proteins not removed in the sample preparation procedure. Figure 2B shows the close resemblance between the product ion spectrum of *m*/*z* 507.29 in sample S4 and T11a in the ricin reference digest.

The LC-MS/MS technique is one of the few techniques, which can effectively differentiate ricin from RCA120. Although a wide variety of techniques such as enzyme-linked immunosorbant assay (ELISA), cytotoxicity, Western blots, gel electrophoresis, nuclear magnetic resonance (NMR), lateral flow assay, hemagglutination and mass spectrometry were used to analyze the samples in this EQuATox study, most methods could not differentiate between ricin and RCA120. LC-MS/MS techniques were the most effective at correctly differentiating high concentrations of ricin and RCA120 with success rates of 79%–87% when looking at sample S2 with a high concentration of RCA120 and sample S6 with a high concentration of ricin as seen in Table 3 and described in a separate publication in this special issue [14].

**Table 3:** Success rates for various methods of differentiation of ricin and RCA120 of the two highest concentration samples.

Sample	Principle	Mean Success Rate
S2	Mass spectrometry	79%
S2	Functional method	57%
S2	Immunological method	43%
S6	Mass spectrometry	87%
S6	Immunological method	71%
S6	Functional method	43%

While the EQuATox program did not ask for differentiation beyond ricin and RCA120, LC-MS/MS methods have proven effective at an extended level of differentiation. Differentiation between ricin and RCA120 or between ricin D and ricin E can be very important for forensic or epidemiological purposes, and such a distinction is often necessary for law enforcement reasons. Both ricin isoforms were present in the EQuATox spiking material and ricin D-specific peptides T14b-T16b disulfide connected peptide, T18b, T19b and T20b were detected (Table 1). However, differentiation of ricin E in the presence of the D isoform and RCA120 is complicated by the complete overlap of the amino acid sequence with either the D isoform or with RCA120. The presence of the E isoform can be determined by quantification of the sum of ricin E and RCA120 using the E/RCA120 shared peptides, subtracted by the amount of RCA120 using the RCA120-specific peptides and calibration samples of a purified RCA120 standard. Alternatively, the D and E isoforms can be separated using ion-exchange chromatography [24], so it is possible that this further differentiation could have been made as part of the EQuATox study if it was in question.

## 4. MS and MS/MS Techniques for Analysis of Ricin’s Enzymatic Activity

### 4.1. Importance of Ricin Activity Measurements

As mentioned previously, ricin has a specific *in vivo* enzymatic activity. It depurinates an adenosine which is part of the GAGA tetraloop of 28S ribosomal RNA, thereby halting protein synthesis. Several laboratories have shown that the enzymatic activity of the A-chain of ricin can be monitored by mass spectrometry. Typically, this process involves incubation of the protein of interest with a shortened mimic of 28S ribosomal RNA, which contains the GAGA tetraloop. Ricin will depurinate the GAGA tetraloop, resulting in the release of free adenine and a decrease in mass of the 28S ribosomal RNA mimic. This assessment of the functionality of the A-chain of ricin is important for anyone desiring to understand the health threat of ricin. However, there are a number of ribosome inactivating proteins (including RCA120) with the same enzymatic activity of ricin, so such detection methods work best with supplementation or in combination with another method to address specificity and/or by the use of functionally blocking antibodies [29].

### 4.2. MS Analysis of Ricin Activity Measurements

**Figure 3:**
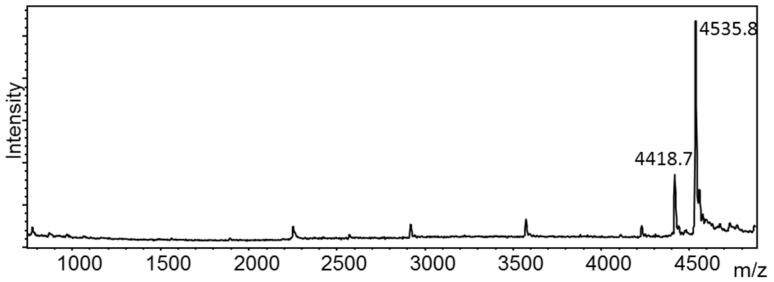
Mass spectrum of the reaction of an RNA substrate with ricin; the peak at *m*/*z* 4535.8 corresponds to the mass of the intact substrate and the peak at *m*/*z* 4418.7 corresponds to depurination of the RNA substrate by ricin.

Although ricin may be subject to denaturation, especially in food samples, the decrease in mass of the 28S ribosomal RNA mimic can be monitored by mass spectrometry, allowing for an accurate determination of the enzymatic activity of the A-chain of ricin, as seen in Figure 3. One successful example of this method involves a 2009 report in which ricin’s enzymatic activity was detected using MALDI-TOF MS to detect the depurination of a DNA mimic of 28S ribosomal RNA [26]. This method was also used to detect the enzymatic activity of ricin in swabs from a public health investigation [27], as well as to detect enzymatically active ricin spiked into a variety of beverages with a reported limit of detection of approximately 64 ng/mL [21]. This method was later adapted for analysis of ricin in a shorter timeframe [30]. Although this analysis was not used in the EQuATox study, it remains a viable alternative for evaluation of the enzymatic activity of ricin provided that the specificity limitations of the assay are well understood.

### 4.3. MS/MS Analysis of Ricin Activity Measurements

Upon depurination of a 28S ribosomal mimic by ricin, free adenine is released from the GAGA tetraloop. Rather than monitoring the mass shift of the RNA mimic, this release of free adenine can be monitored by MS/MS techniques. This technique was first reported in 2007, using a triple quadrupole mass spectrometer measuring adenine and its specific fragment ions [31]. This technique resulted in the ability to detect ricin at levels as low as 0.1 ng/mL following concentration of the toxin from 0.5 mL of sample [31]. Recently, this method was used to examine the enzymatic activity of ricin in eighteen cultivars from *R. communis* [22]. The approach was used by at least one laboratory as part of the EQuATox study, and the ricin and/or RCA120 protein were found in samples S2, S3, S4, S5, S6, S8 and S9 as seen in Table 1. Sample S7 contained the lowest ricin concentration of all the samples, and due to sample volume limitations, ricin activity was not observed in that sample.

An optional challenge of the EQuATox project was to rank the functional activity of three of the samples, S1, S3 and S5, using the designations of “highest”, “intermediate” and “lowest” functional activity. Through analysis of the released adenine, LC-MS/MS in at least one laboratory correctly identified sample S1 as the least in functional activity followed by sample S5 as intermediate functional activity and then sample S3 as highest functional activity. All three of these samples consisted of the same buffer matrix, but contained either no toxin (S1), 445 μg/L of RCA120 (S5), or 504 μg/L of ricin (S3). Even though the concentrations of toxin in samples S3 (containing ricin) and S5 (containing RCA120) were similar, their functional activities were correctly assigned. This analysis also used immunoaffinity enrichment with antibodies to both ricin and RCA120.

## 5. Addition of Affinity Techniques Assists MS and MS/MS Analysis of Ricin

Although mass spectrometric measurements of ricin can yield useful information, a purification and enrichment step prior to MS or MS/MS analysis assists in controlling unwanted background interferences and lowers the limit of detection. The increase in the concentration of ricin in the sample makes it possible to use MS identification techniques to unambiguously confirm results obtained by highly sensitive ELISA and activity measurements in extremely dilute samples.

### 5.1. Immunoaffinity as a Purification Technique

Addition of immunoaffinity prior to ricin mass spectrometric analysis was first reported in 2007 where the authors used this technique prior to LC-MS/MS analysis of adenine released as a result of ricin’s enzymatic activity [31]. Since then, many studies have reported on the use of immunoaffinity prior to mass spectrometric analysis, including MS of the toxin itself [19,32], MS/MS of the toxin [21,22,26,27], MS of ricin’s activity [21,26,27] and MS/MS of ricin’s activity [22]. Several successful EQuATox mass spectrometric analyses used immunoaffinity capture prior to mass spectrometry, such as the data presented in Table 1. Both the purification and concentration of the ricin by immunoaffinity was critical to these mass spectrometric measurements, especially in complex sample matrices.

### 5.2. Carbohydrate Affinity as a Purification Technique

Other techniques for purification/concentration prior to mass spectrometric analysis of ricin involved the ability of ribosome-inactivating proteins such as ricin to bind galactose-terminated glycolipids and glycoproteins on the cell surface. Galactose affinity has commonly been used for large-scale purification of ricin and other RIP-II toxins from plant material [33,34]. Adaption to analytical scale purification was reported in 2011, with the use of lactose immobilized to monolithic silica to extract ricin prior to MS/MS analysis [35]. Another report in 2015 used a galactose-terminated ligand bound to chromatographic resin prior to MS/MS analysis of ricin and other ribosome-inactivating proteins [28]. One laboratory successfully used this affinity technique in all of the EQuATox samples except for the sample containing spiked milk as the lactose content, which prevents lectin binding and requires the use of immunoaffinity purification. Thus, for most sample matrices, galactose affinity remains a viable and less expensive alternative to immunoaffinity and has the advantage of enrichment of multiple ribosome-inactivating proteins.

## 6. Quantification of Ricin and Its Enzymatic Activity

### 6.1. Transforming a Qualitative MS Assay into a Quantitative Assay

Mass spectrometric assays for ricin can be transformed from qualitative to quantitative through the addition of an internal standard into the samples and into a calibration curve, containing known amounts of ricin. In most cases, the internal standard is an isotopically-labeled version of the analyte: either an isotopically-labeled tryptic peptide in the case of MS or MS/MS analyses of the ricin toxin or an isotopically-labeled version of adenine in MS/MS assays to detect ricin’s enzymatic activity. The use of an isotopically-labeled version of the native analyte ensures that the chemical properties of the internal standard which determine chromatographic retention time and ionization potential are the same as the native analyte, yet the internal standard can easily be differentiated from the native analyte due to the increase in mass.

### 6.2. Quantification of Ricin by MS/MS

The amount of ricin in the EQuATox samples was reported using MS/MS on the toxin itself by one laboratory. The qualitative analyses of the EQuATox samples showed that samples S3, S4, S6, S8 and S9 contained ricin. Table 4 lists the concentrations of ricin, using a targeted LC-MS/MS method with immunoaffinity purification and peptides underlined in Figure 1 and listed in Table 1, found in samples S3, S4, S6, S7, S8 and S9 along with the nominal concentrations. One measure of accuracy is known as a z-score, which indicates how many standard deviations a particular value is from the mean. Z-scores in the range of −2 to +2 are considered to be acceptable. The z-scores are also listed in Table 4, and four of the six ricin measurements (Table 4) were within this acceptable range, and only one score lay outside of +/−3, indicating room for improvement. The one measurement in the unacceptable range was for the one solid sample, the fertilizer, and it is likely that an error in the creation of a liquid extract from the fertilizer caused the quantification error. Additionally, the one measurement outside of the −2 to +2 range was below the limit of detection. Nonetheless, the results are quite good taking into account that different reference materials and a technically different method were used to compare to the reference materials and the method used in the organizing laboratory, respectively.

**Table 4:** Quantitative results for MS/MS analysis of EQuATox samples for ricin.

Sample	Nominal Conc.	Unit	Obs Conc. #1	Obs Conc. #2	*Z*-Score
S3	504	μg/L	590	693	0.89
S4	473	μg/L	641	641	1.85
S6	589508	μg/L	507,500	526,600	−0.48
S7	0.414	μg/L	0.22	0.10	−2.5
S8	484	μg/L	497	659	0.54
S9	306	mg/kg	15.3	12.5	−3.66

### 6.3. Quantification of RCA120 by MS/MS

Samples S2, S5 and S9 contained RCA120, and one laboratory attempted quantification of the RCA120 by mass spectrometric analysis, using the peptides underlined in Figure 1 and listed in Table 2. The results of the analyses were that sample S2 contained an average of 776,000 μg/L, sample S5 contained 997 μg/L and sample S9 contained an average of 4 mg/kg of RCA120. The actual concentrations were revealed to be 572,851 μg/L for sample S2, 445 μg/L for sample S5 and 42 mg/kg for sample S9. Only the measurement for sample S2 had a z-score within +/−2. This is likely due in part to the emphasis of assays on ricin rather than RCA120, as fewer ions were used to monitor RCA120 as compared to ricin, and the general optimization of the assay for the quantification of ricin. Another source of error could be the difficulty in obtaining reference materials of purified ricin, which do not contain RCA120 and *vice versa*, as there are no known cultivars, which produce either toxin exclusively. The purity of reference materials used in the EQuATox study was >97% for ricin and >99% for RCA120 [36]. Other commercial sources of ricin and RCA120 such as the material used for the calibration curves contain higher levels of contamination from RCA120 and ricin [20]. Therefore, methods, which are sensitive, could yield a positive result for RCA120, for instance, particularly in the presence of a high concentration of ricin. Highly accurate and reproducible quantification requires that a reliable and well-characterized reference standard be employed.

## 7. Summary

Through the EQuATox study, it has been demonstrated that mass spectrometric measurements of ricin play a vital role in correct identification of ricin in complex matrices. Some mass spectrometric measurements of ricin serve as accessory methods which, when used in combination with other methods, can yield information helpful in determining the presence of ricin either through a molecular weight measurement designed to differentiate ricin from RCA120 or through an enzymatic activity measurement of a ribosome-inactivating protein as seen in Figure 3. Other mass spectrometric measurements, such as MS/MS measurements, can stand alone as a definitive identification of ricin, proving the existence of the unique amino acid sequence of ricin.

In analyses with limited sample volume and the desire to perform multiple mass spectrometric measurements, a valid approach would be to divide each sample into separate aliquots for enzymatic activity measurements and structural measurements, perhaps putting more emphasis on the enzymatic activity measurements for public health purposes or highlighting the structural measurements for forensic purposes. For structural measurements to be used as definitive identification of ricin, MS/MS detection of two peptides specific and unique to ricin, such as some of the peptides listed in Table 1, can be used for positive detection.

EQuATox is the first international program to investigate qualitative and quantitative results for an assortment of ricin detection methods using well-characterized analytical standards. The results from EQuATox will become an important basis for international discussions on the exact needs for ricin quantification for security and public health and what reference standards are necessary in order to meet patient, public health and law enforcement needs. Mass spectrometry can play a critical role in the correct identification of ricin in complex matrices. With its ability to yield the functional activity measurements critical for public health needs and its ability to accurately differentiate between ricin and RCA120 needed for forensics reasons, mass spectrometry is a useful tool for ricin detection encompassing a wide variety of detection goals.

## 8. Experimental Section

### 8.1. Targeted MS/MS Qualitative and Quantitative Analysis

Targeted MS/MS qualitative analysis proceeded as described previously [22]. Essentially, magnetic beads coated with antibodies to ricin and RCA120 were added to the sample and allowed to bind toxin if present. Following bead washing, the beads were digested with trypsin and the subsequent peptides were analyzed by LC-MS/MS on a triple quadrupole mass spectrometer, monitoring for peptides underlined in Figure 1 and listed in Table 1. Transitions monitored were previously described [22]. Quantitative analyses were performed in a similar fashion using isotopically-labeled tryptic peptides and transitions as described earlier [22].

### 8.2. Accurate Mass LC-MS and MS/MS Qualitative Analysis

Non-targeted LC-MS and MS/MS analyses were performed as described previously [28]. Briefly, sample aliquots (10–200 μL depending on quantification results obtained by ELISA) were purified and concentrated by galactose affinity chromatography. Galactose-binding proteins were digested with trypsin without prior reduction and alkylation. Sample S4 was suspected to contain milk, in which lactose interferes with the galactose affinity purification. Therefore, aliquots of S4 were immunopurified using a biotinylated polyclonal antibody to ricin and RCA120 coupled to Dynabeads Streptavidin T-1 magnetic beads.

The digests were analyzed by high-resolution accurate mass nanoLC-MS on a QTOF instrument (Waters Corporation, Milford, MA, USA), and the retention times and masses of the peptides were compared with those pf ricin and RCA120 reference digest. The identity of diagnostic peptides was confirmed by nanoLC-MS/MS analysis using time-scheduled precursor ion selection and comparison with the corresponding peptides in the reference digests.

### 8.3. MS/MS of Enzymatic Activity of Ricin

The enzymatic activity of ricin was examined as described previously [22,31]. Basically, following immunocapture of ricin or RCA120 from the sample and bead washing, the toxin on the beads was allowed to interact with RNA substrate CGCGCGAGAGCGCG or DNA substrate GCGCGAGAGCGC, resulting in the release of free adenine in the presence of ricin or RCA120. The presence of free adenine was monitored using triple quadrupole mass spectrometry using the transitions of *m*/*z* 136 to 119, 136 to 92 and 136 to 65 as described previously [22,31]. Quantification of the enzymatic activity of ricin and RCA120 was also accomplished as previously described [22,31] using either ^15^N_2_-1,3-adenine or ^13^C-labeled adenine as an internal standard.
